# Early Dinner Improves the Glycemic Profile in Habitual Late Eaters With Uncontrolled Type 2 Diabetes Mellitus in the Short Term

**DOI:** 10.7759/cureus.59504

**Published:** 2024-05-02

**Authors:** Yash V Chauhan, Jugal V Gada, Sukirti Misra, Charushila B Dhole, Anagha V Palekar, Premlata K Varthakavi, Nikhil M Bhagwat

**Affiliations:** 1 Department of Endocrinology, Topiwala National Medical College and Bai Yamunabai Laxman Nair Charitable Hospital, Mumbai, IND; 2 Department of Dietetics, Topiwala National Medical College and Bai Yamunabai Laxman Nair Charitable Hospital, Mumbai, IND

**Keywords:** insulin resistance, glycemic control, continuous glucose monitoring system, type 2 diabetes mellitus, late dinner, early dinner

## Abstract

Background

Late dinner (LD) can worsen the glucose profile in type 2 diabetes (T2D). We assessed the short-term effect of early dinner (ED) on glycemic control in habitual late eaters with uncontrolled T2D.

Methodology

This interventional, single-arm, within-group trial recruited 10 habitual late eaters with uncontrolled T2D (glycosylated hemoglobin: 7-9% and either fasting plasma glucose (FPG): ≥140 mg/dl or post-prandial plasma glucose: ≥200 mg/dl). They had their usual LD (beyond 22:00 hours) on Days 0-3 and ED (before 20:00 hours) on Days 4-10. Continuous glucose monitoring system (CGMS) parameters, two-hour post-dinner, and fasting (10-hour post-dinner) investigations were analyzed. Bedtime hunger was assessed using a Labeled Magnitude Satiety Scale.

Results

The mean dinner time was reduced from 22:28 hours to 19:29 hours. CGMS revealed that ED lowered the 10-hour post-dinner incremental area under the curve (22,587.9 ± 5,168.3 mg/dl×mins vs. 15,886.3 ± 4,288.7 mg/dl×mins, P < 0.002), 10-hour post-dinner average blood glucose (ABG) (137.5 ± 9.3 mg/dl vs. 125 ± 7.9 mg/dl, P < 0.002), 24-hour ABG (132.2 ± 7.5 mg/dl vs. 124.8 ± 5.4 mg/dl, P = 0.037), and night mean amplitude of glucose excursion (83.7 ± 5.8 mg/dl vs. 69.3 ± 7.5 mg/dl, P = 0.027). ED also reduced FPG (119.8 ± 7.3 mg/dl vs. 105.2 ± 5.7 mg/dl, P = 0.015), fasting insulin (15.0 ± 4.3 µIU/ml vs. 9.7 ± 2.7 µIU/ml, P < 0.002), and HOMA-IR (4.36 ± 1.2 vs. 2.56 ± 0.79, P < 0.002). Post-dinner glucose, insulin, and inflammatory markers were unchanged. Bedtime hunger increased significantly on Days 4 and 5 but returned to baseline by Day 10.

Conclusions

A simple modification of dinner time in habitual late eaters with uncontrolled T2D improves FPG, glycemic control, and insulin resistance in the short term.

## Introduction

Late dinners (LDs) are frequently the main meal of the day, especially in urban areas with a high burden of type 2 diabetes (T2D) [1-3]. Major international recommendations do not include meal times but emphasize a comprehensive, individualized approach [4]. LD with more calories later in the day enhances the risk of metabolic derangements because sleep reduces the basal metabolic rate, leading to impaired handling of ingested substrates and a lower rate of nutrient oxidation [5-7]. A late meal also worsens nighttime glucose tolerance, increasing carbohydrate digestion and absorption, which may persist into breakfast, worsening the total glucose profile [8,9]. Insulin resistance (IR) among healthy individuals is reported to be lowest in the morning and highest in the evening, and this diurnal periodicity in glucose tolerance is robustly regulated by the circadian timing system, independent of other influences [10,11]. Furthermore, diet-induced thermogenesis is more than 2.5 times higher in the morning than in the evening, resulting in more significant glucose peaks with a heavier meal in the evening [12].

This pattern of IR is reversed in T2D, with glucose levels demonstrating a night-to-morning elevation increasing in the early hours of the morning and a reduction in IR during the waking day [13,14]. Increased hepatic glucose output contributes to this “dawn phenomenon” [15]. In subjects with T2D, late-night meals are associated with poor glycemic control, and the postprandial hyperglycemia was alleviated by eating a divided dinner [3,16,17]. There is very little data to establish the influence of dinner timing on glycemic management, especially in Indian subjects whose meals are predominantly high in carbohydrate content [18].

We hypothesized that an early dinner (ED) would improve overall glycemic control by reducing the nighttime increase in IR. We conducted a study using a continuous glucose monitoring system (CGMS) to determine the effect of ED on glycemic control in habitual late eaters with uncontrolled T2D.

The present study evaluated the short-term effect of ED on 24-hour average blood glucose (ABG), 10-hour post-dinner incremental area under the curve (iAUC) of glucose, 10-hour post-dinner ABG as measured by CGMS, and fasting plasma glucose (FPG) levels in habitual late eaters with uncontrolled T2D. We additionally assessed the effect of ED on measures of glycemic variability (mean amplitude of glucose excursion (MAGE) and coefficient of variation (CV)), post-dinner plasma glucose levels, fasting and post-dinner insulin, lipid profile, inflammatory markers (high-sensitivity C-reactive protein (hsCRP), interleukin-6 (IL-6)), and markers of oxidative stress (malondialdehyde (MDA) levels).

## Materials and methods

This study was conducted at Topiwala National Medical College and Bai Yamunabai Laxman Nair Charitable Hospital, Mumbai, India.

Participants

This study was approved by the institutional ethics committee and review board (ECARP/2020/59). Participants were recruited after written informed consent from a diabetes clinic in a tertiary care hospital in Mumbai, India, between July 2021 and March 2022.

We included patients with T2D aged ≥18 years who ate their last meal beyond 22:00 hours and were on a stable modified diet and antidiabetic medications for the preceding three months. They had glycosylated hemoglobin (HbA1c) values of 7-9% and either FPG ≥140 mg/dl or post-lunch plasma glucose ≥200 mg/dl. Only patients taking metformin and/or dipeptidyl peptidase-4 inhibitors were included. We excluded night shift workers, those with irregular sleep timings, type 1 diabetes mellitus, T2D with insulin or other treatment, history of alternate medications, glucocorticoid use, impaired renal function (estimated glomerular filtration rate <60 ml/min), impaired liver function (elevated transaminase levels ≥3 times upper limit of normal or known chronic liver disease), cardiovascular disease, obstructive sleep apnea, pregnancy or lactation, any other acute or chronic systemic illness, and psychiatric disorders, including illicit drug and alcohol abuse.

Study design and procedure

This was a prospective single-center, interventional, single-arm, within-group trial. Participants were screened for eligibility criteria two weeks prior to the initiation of testing. Diet, medication, demographic and sleep history, physical examination, and baseline investigations were documented. All participants were on a consistent diet for at least the preceding three months, modified by a dietician, with an intake ranging from 1,400 to 1,800 kcal per day.

LD was defined as the last meal for the day beyond 22:00 hours, and ED was defined as the last meal for the day before 20:00 hours. No food, except water, was permitted thereafter. The participants carried their usual dinner on the day of the tests.

**Figure 1:**
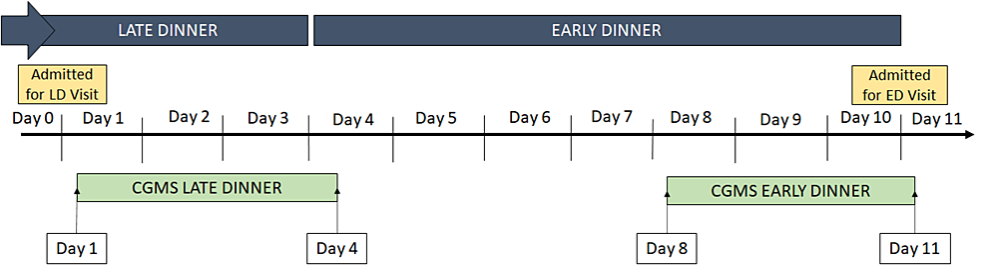
Study protocol The patients ate LD beyond 22:00 hours on Days 0-3 and ED before 20:00 hours on Days 4-10. CGMS values were considered from Days 1-4 for LD and Days 8-11 for ED. CGMS, continuous glucose monitoring system; ED, early dinner; LD, late dinner

On the test day (Day 0), eligible participants were admitted to an indoor facility (Figure 1). A CGMS (FreeStyle Libre Pro Sensor Flash Glucose Monitoring, Abbott Healthcare Private Limited, Green Oaks, Illinois, USA) was applied. All patients were provided with a self-monitoring capillary glucose kit (OneTouch Select Plus, LifeScan Inc., Zug, Switzerland) and strips to monitor glucose for 10 days, providing baseline values for CGMS. Written instructions were provided to monitor fasting, pre-dinner, and two-hour post-dinner glucose. Each participant was instructed to maintain a food diary, which included meal timing and composition, physical activity, and sleep timing.

A Hunger-Satiety Scale (Labeled Magnitude Satiety Scale (LMSS)), as developed by Cardello et al. [19] and modified by Solah et al. [20], was provided and explained to the participants to be filled out at their usual bedtime. The labels were numbered 1 through 11 to facilitate the participant’s note-taking, with 1 denoting “Greatest Imaginable Hunger” and 11 denoting “Greatest Imaginable Fullness.” Relevant permission for using the scale was obtained, and the scale was translated into local languages and cross-translated by independent translators.

The participants ate their usual dinner beyond 22:00 hours (LD visit). Post-dinner and fasting blood samples were collected. The patient was then discharged. Thereafter, the patient ate LD for three days (Days 1-3) and ED for the next seven days (Days 4-10). The participant followed the same routine meal pattern, portion, composition, physical activity status, and sleep pattern throughout the study, except for dinner timings. The participants were followed up on a daily telephonically to ensure compliance.

They were then readmitted on Day 10 and ate their usual dinner before 20:00 hours (ED visit). Post-dinner and fasting blood samples were collected. The CGMS data was collected and removed after 08:00 hours on Day 11.

Blood samples

For both visits, post-dinner blood samples were collected two hours after dinner, and fasting samples were collected 10 hours after dinner. Blood samples were collected in clot activator tubes for serum investigations and fluoride tubes for plasma glucose. The plasma glucose was processed immediately. Blood in clot activator tubes was allowed to clot for 30 minutes and centrifuged at 2,000 rpm for 10 minutes. The serum was separated using sterile pipettes and stored at -80 °C until further processing.

Laboratory investigations

HbA1c was processed using high-performance liquid chromatography (Bio-Rad D10 analyzer, Bio-Rad Laboratories, Inc., Hercules, California, USA; inter-assay CV: 1%, intra-assay CV: 0.8%). Plasma glucose was estimated using the hexokinase method (Roche COBAS 6000, F. Hoffmann-La Roche Ltd., Basel, Switzerland; inter-assay CV: 1%, intra-assay CV: 0.8%). Serum insulin was estimated by electrochemiluminescence immunoassay (Beckman Coulter Access-2, Beckman Coulter, Inc., Brea, California, USA; inter-assay CV: 3.5%, intra-assay CV: 2%). The homeostasis model of assessment of IR (HOMA-IR) was calculated using a standard formula ((FPG (mg/dL) × fasting serum insulin levels (µIU/ml))/405) [21]. hsCRP was analyzed by immunoturbidimetry/nephelometry (Abbott Architect, Abbott Healthcare Private Limited; inter-assay CV: 0.5-2.4%, intra-assay CV: 0.7-4%). IL-6 was estimated using an electrochemiluminescence immunoassay (Roche COBAS e411, F. Hoffmann-La Roche Ltd.; inter-assay CV: 8.5%, intra-assay CV: 6%). The thiobarbituric acid (TBA) reactive substances method was used to determine serum MDA levels. MDA is formed due to lipid peroxidation and reacts with TBA under high temperatures (90-100°C) and acidic conditions. The reaction yielded a pink MDA-TBA adduct and was measured spectrophotometrically at 530 nm.

CGMS

The FreeStyle Libre Pro Flash Glucose Monitoring System is a blinded professional CGMS that records glucose values every 15 minutes for up to 14 days. Data from CGMS was exported using the manufacturer’s software. CGMS values were considered for LD on Days 1-3 and for ED on Days 8-10 (Figure 1). iAUC (expressed in mg/dl×mins) was calculated above the baseline pre-dinner value for 10-hour post-dinner. The iAUC was further divided into 0-180 minutes post-dinner (three-hour post-dinner iAUC), 180-360 minutes post-dinner (midnight iAUC), and 360-600 minutes post-dinner (early morning iAUC). ABG was calculated for every 24 hours (24H ABG) from 08:00 hours to 08:00 hours for LD (Days 1-4) and ED (Days 8-11) (Figure 1). The 10-hour post-dinner ABG was calculated for Days 1-3 for LD and Days 8-10 for ED.

MAGE and CV were considered measures of glycemic variability [22] and were calculated separately for 24 hours (24H MAGE and 24H CV, respectively) and 10-hour post-dinner (Night MAGE and Night CV, respectively).

Statistical analysis

The sample size was calculated using data from Imai et al. [16]. Expecting a 6.99% fall in mean plasma glucose values between early and LD in patients with T2D and assuming 95% confidence intervals and 80% power, we found a minimum sample size of 6.653 patients. A total of 10 patients were studied to account for dropouts.

All analyses were performed using Microsoft Excel 2019 (Microsoft Corporation, Redmond, Washington, USA), GraphPad Prism 9.0 (GraphPad Software, LLC, San Diego, California, USA), and IBM SPSS Statistics for Windows, Version 28.0 (Released 2021; IBM Corp., Armonk, New York, USA). All values are expressed as mean ± SEM. iAUC was calculated using an algorithm developed on MS Excel using the trapezoidal method and cross-checked on GraphPad Prism 9.0. The ABG was calculated as the average of all glucose readings at the given time. MAGE and CV were determined using EasyGV software version 9.0.R2 (EasyGV, University of Oxford).

For each quantitative variable, a test for normality was performed using the Shapiro-Wilk test. If the data were normally distributed, paired t-tests were applied between the two means. If the data was not normally distributed, the Wilcoxon matched-pairs signed-rank test was applied between the two medians. A P-value of <0.05 was considered significant.

## Results

Baseline patient characteristics

Ten participants (three females and seven males) with T2D and baseline demographics, as shown in Table 1, were recruited. All patients had a fixed sleep, diet, and exercise pattern. There was no habitual breakfast skipper.

**Table 1:** Baseline demographic characteristics of participants Gender and oral antidiabetic medications are expressed as N (%). All continuous data are expressed as mean ± SEM. DPP4i, dipeptidyl peptidase-4 inhibitors; FPG, fasting plasma glucose; HbA1c, glycosylated hemoglobin

Variable	N = 10
Gender	
Males	7 (70%)
Females	3 (30%)
Age (years)	52.5 ± 4.46
Duration of diabetes mellitus (years)	4.225 ± 1.47
FPG (mg/dl)	137 ± 4.84
Post-lunch plasma glucose (mg/dl)	211.9 ± 7.7
HbA1c (%)	7.54 ± 0.1
BMI (kg/m^2^)	28.14 ± 0.82
Usual dinner time (hours:minutes)	22:33 ± 00:14
Usual sleep onset time (hours: minutes)	23:42 ± 00:14
Usual sleep offset time (hours:minutes)	06:57 ± 00:28
Systolic blood pressure (mmHg)	121.4 ± 3.68
Diastolic blood pressure (mmHg)	81 ± 2.5
Oral antidiabetic medications	
Metformin	10 (100%)
DPP4i	3 (70%)

Transition to an ED

**Figure 2:**
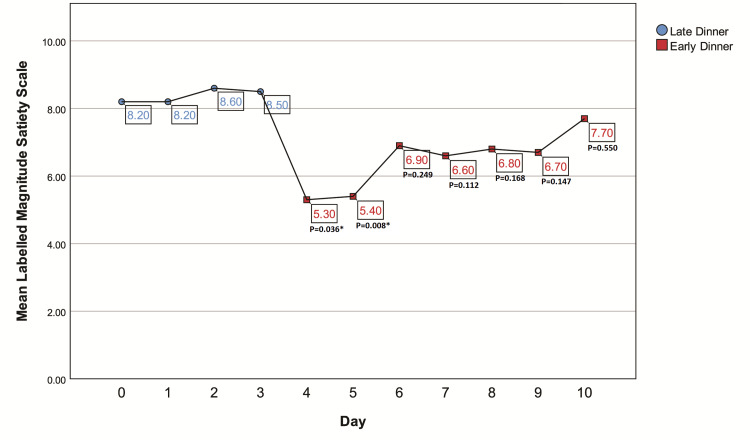
Mean LMSS score on Days 0 to 10, noted by the participants at their usual bedtime Days 0-3 are LDs, and Days 4-10 are EDs. We found a significantly lower score on Days 4 and 5 (5.3 and 5.4 on Days 4 and 5, respectively, P < 0.05 as compared to Day 0), indicating greater hunger at bedtime that gradually increased to baseline (7.7 on Day 10, P = 0.550 as compared to Day 0). * P-value <0.05 as compared to Day 0 ED, early dinner; LD, late dinner; LMSS, Labeled Magnitude Satiety Scale

The patients had LD on Days 0-3 at an average time of 22:28 hours and ED on Days 8-10 at an average time of 19:29 hours. Compliance with dinner timings was 100%, as cross-checked by CGMS readings. Meal composition revealed equal calorie intake throughout the study period (mean calorie intake: 1,690 ± 120.4 kcal for LD vs. 1,687 ± 120.1 kcal for ED; P = 0.24). Sleep onset and offset time were also not different (mean sleep onset: 23:41 hours for LD vs. 23:39 hours for ED; mean sleep offset: 06:48 hours for LD vs. 06:47 hours for ED). The mean LMSS at bedtime was significantly lesser on Days 4 and 5 (5.3 and 5.4, respectively) as compared to Day 0 (8.2); however, it increased gradually to baseline on Day 10 (7.7) (P = 0.550 for Day 10 compared to Day 0) (Figure 2).

Glucose, insulin, and lipid profile values

**Figure 3:**
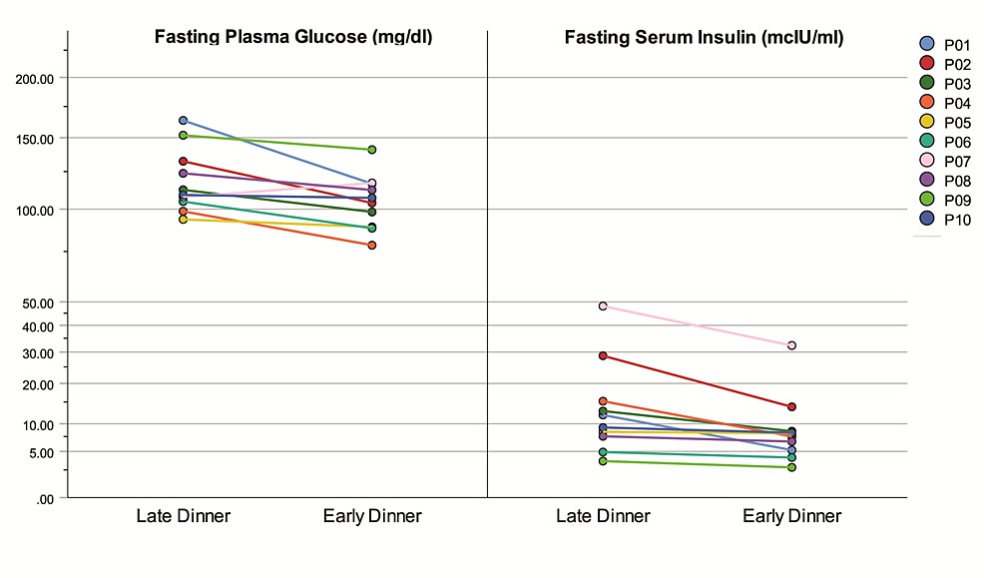
FPG (left panel) and fasting serum insulin levels (right panel) for individual patients There was a significant and consistent reduction in FPG (in nine of 10 patients) and fasting insulin levels (in 10 of 10 patients) between LD and ED. We noted a difference of 14.59 mg/dl in mean FPG levels and 5.33 µIU/ml in mean fasting serum insulin levels between the two visits. ED, early dinner; FPG, fasting plasma glucose; LD, late dinner

Two-hour post-dinner plasma glucose and insulin values were higher, but not significantly higher, in LD than in ED. ED lowered FPG significantly by 14.59 mg/dl and fasting insulin levels by 5.33 µIU/ml (Figure 3). Paralleling the reduction in FPG and serum insulin levels, HOMA-IR also reduced significantly (4.36 ± 1.2 on LD vs. 2.56 ± 0.79 on ED, P < 0.002) (Table 2). The lipid profile showed significant reductions in fasting and two-hour post-dinner total cholesterol during ED than LD. Triglyceride levels were also lower, but not significantly, during ED than LD (Table 2).

**Table 2:** PG, serum insulin, and lipid profile values between the two visits All values are expressed as mean ± SEM. * The P-value is significantly lower in ED than in LD. ED, early dinner; F, fasting; FPG, fasting plasma glucose; HOMA-IR, homeostasis model of assessment of insulin resistance; LD, late dinner; PD, two-hour post-dinner; PG, plasma glucose

Parameter	LD	ED	P-value
FPG (mg/dl)	119.81 ± 7.3	105.22 ± 5.69	0.015*
PD PG (mg/dl)	152.24 ± 14.17	143.83 ± 1.12	0.65
Serum F insulin (mIU/ml)	15.04 ± 4.3	9.71 ± 2.68	<0.002*
Serum PD insulin (mIU/ml)	35.8 ± 12.46	34.89 ± 8.64	0.695
HOMA-IR	4.36 ± 1.2	2.56 ± 0.79	<0.002*
Serum F total cholesterol (mg/dl)	162.8 ± 13.37	145.6 ± 13.09	0.028*
Serum PD total cholesterol (mg/dl)	159.3 ± 12.6	145.7 ± 11.53	0.049*
Serum F triglycerides (mg/dl)	157.5 ± 24.69	129.2 ± 19.85	0.117
Serum PD triglycerides (mg/dl)	179.8 ± 28.57	157 ± 23.97	0.169

CGMS, iAUC, ABG, and glycemic variability

**Figure 4:**
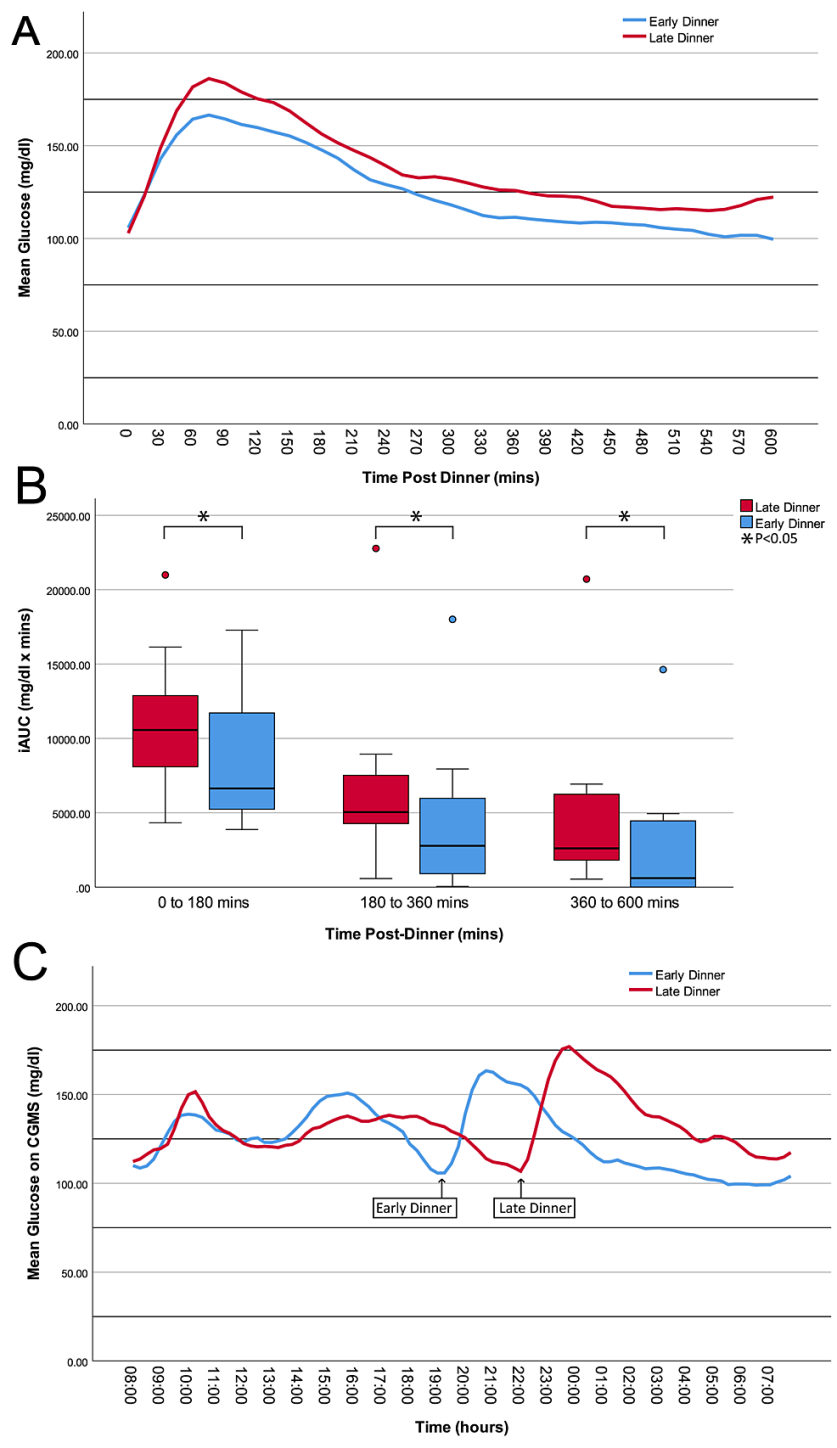
CGMS readings for three days of LD vs. ED (A) depicts mean CGMS readings for three days for 10-hour post-dinner (Days 1-3 for LD and Days 8-10 for ED). We found significant reductions in the 10-hour post-dinner iAUC and 10-hour post-dinner ABG between LD and ED (P < 0.002 for both). (B) shows that after dividing iAUC into three-hour post-dinner (0-180 minutes), midnight (180-360 minutes), and early morning (360-600 minutes), there was a significant reduction of iAUC in all three time intervals (P < 0.05 for all time intervals). (C) depicts mean 24-hour CGMS readings on LD vs. ED days (08:00 hours on Day 1 to 08:00 on Day 4 for LD and 08:00 hours on Day 8 to 08:00 hours on Day 11 for ED). We found a significant reduction in the 24H ABG between LD and ED. * P-value <0.05 CGMS, continuous glucose monitoring system; ED, early dinner; LD, late dinner

Ten-hour post-dinner CGMS readings for three days showed a lower spike in ED that persisted in lower fasting glucose (Figure 4A). Although the pre-dinner glucose was comparable (102.9 ± 3.89 mg/dl for LD vs. 105.77 ± 5.16 mg/dl for ED, P = 0.520), the iAUC for 10-hour post-dinner glucose was significantly lower in ED as compared to LD (Table 3). The iAUC values calculated separately for time intervals of three-hour post-dinner, midnight, and early morning showed significant reductions in all three time intervals during ED as compared to LD (P 0.017, 0.021, and 0.013, respectively; Table 3 and Figure 4B). In addition, 24-hour CGMS readings showed a smaller and earlier post-dinner peak (Figure 4C). Both 10H and 24H ABG were significantly lower during ED than LD (Table 3). Night MAGE was significantly lower during ED than LD; however, neither 24H MAGE nor Night nor 24H CV were significantly different during ED compared to LD.

**Table 3:** Mean CGMS values for three days for LD (Days 1-3) and ED (Days 8-10) All values are expressed as mean ± SEM. * The P-value is significantly lower in ED than in LD. 10H, 10-hour post-dinner; 24H, 24 hours; ABG, average blood glucose; CGMS, continuous glucose monitoring system; CV, coefficient of variation; ED, early dinner; iAUC, incremental area under the curve; LD, late dinner; MAGE, mean amplitude of glucose excursion

Parameter	LD	ED	P-value
iAUC for 10-hour post-dinner (mg/dl×mins)	22,587.88 ± 5,168.34	15,886.29 ± 4,288.7	<0.002*
iAUC 0-180 (mg/dl×mins)	11,206.14 ± 1,492.62	8,466.27 ± 1,348.47	0.017*
iAUC 180-360 (mg/dl×mins)	6,571.07 ± 1,985.72	4,563.18 ± 1,718.15	0.021*
iAUC 360-600 (mg/dl×mins)	4,810.67 ± 1,887.72	2,856.84 ± 1,442.61	0.013*
10H post-dinner ABG (mg/dl)	137.45 ± 9.31	125.06 ± 7.88	<0.002*
24H ABG (mg/dl)	132.22 ± 5.41	124.84 ± 7.47	0.037*
Night MAGE (10H post-dinner) (mg/dl)	83.72 ± 5.84	69.27 ± 7.45	0.028*
Night CV (10H post-dinner) (%)	21.70 ± 0.86	22.63 ± 2.55	0.72
MAGE 24H (mg/dl)	92.39 ± 4.95	90.10 ± 6.13	0.64
CV 24H (%)	26.15 ± 0.94	28.10 ± 1.8	0.24

Inflammatory markers and markers of oxidative stress

Serum hsCRP and IL-6, although reduced in ED compared to LD, did not reach statistical significance. MDA levels did not significantly differ between the two visits (Table 4).

**Table 4:** Inflammatory markers and markers of oxidative stress during LD vs. ED All values are expressed as mean ± SEM. * The P-value is significantly lower in ED than in LD. ED, early dinner; F, fasting; hsCRP, high sensitivity C-reactive protein; IL-6, interleukin 6; LD, late dinner; MDA, malondialdehyde; PD, two-hour post-dinner

Parameter	LD	ED	P-value
F hsCRP	4.09 ± 1.38	3.45 ± 0.79	0.977
PD hsCRP	4.11 ± 1.43	4.08 ± 0.97	0.713
F IL-6	5.90 ± 0.93	4.82 ± 0.93	0.082
PD IL-6	6.31 ± 0.99	4.44 ± 0.58	0.065
F MDA	14.62 ± 0.98	13.82 ± 1.12	0.16
PD MDA	13.57 ± 0.98	14.37 ± 1.24	0.121

## Discussion

This study revealed that solely modifying dinner timings without changing other parameters improves glycemic control, post-prandial glycemic excursions, FPG, nighttime glycemic variability, and IR in T2D patients. The FPG and fasting insulin decreased consistently in almost all patients (Figure 3) during the ED visit compared to the LD visit. Changes in dinner time did not significantly reduce two-hour post-dinner glucose or insulin levels. This result could not be attributed to the prolonged fasting time alone in ED because iAUC significantly reduced in all three time intervals (three-hour post-dinner iAUC, midnight iAUC, and early morning iAUC), and the fasting samples were collected 10 hours after the last meal during both visits. Thus, preponing the dinner time reduced post-dinner glucose excursion and FPG levels independent of the prolonged fasting time interval and was attributable to a reduction in IR, as shown by the significant reduction in HOMA-IR. In healthy populations, IR is highest toward the evening, whereas in T2D, IR worsens throughout the night and is highest during the early morning hours [15]. An explanation for the improvement in IR in our study could be an improvement in this circadian misalignment, resulting in a shift of IR toward natural physiology. Other studies of human and animal subjects involving time-restricted feeding have shown similar improvements in IR by reprogramming glucose metabolism away from gluconeogenesis and toward anabolic pathways [23,24]. Although reducing inflammatory markers could reduce IR, we did not find a significant decrease in them. The higher fasting glucose levels in LD versus ED at 10-hour post-dinner could also be due to increased counter-regulatory hormones during LD and not during ED. However, one study found lower morning cortisol levels in habitual LD eaters than ED eaters, undermining this argument [25].

We also found significant reductions in total cholesterol and nonsignificant reductions in triglyceride levels. Cholesterol biosynthesis predominantly occurs at night between 00:00 and 06:00 hours and follows a 24-hour circadian rhythm [26]. Based on our findings, we hypothesize that eating dinner earlier may lessen the peak of cholesterol production, resulting in lower total cholesterol levels. Gu et al. in 2020 found that an LD had no impact on 10-hour post-dinner fasting triglycerides between an ED and LD similar to ours and that the triglyceride peak was delayed and prolonged, having a peak six hours after the LD [7]. We collected blood samples two-hour post-dinner and may have missed the peak.

Another finding is the significant reduction in the 10-hour iAUC of glucose over the baseline pre-dinner value by 6,701.59 mg/dl×mins (a 29.66% reduction). This is consistent with those previously reported by Sato et al. and Imai et al. [9,17], but lower than that of Imai et al. in 2017, who reported a 4.5 times higher iAUC for T2D patients consuming late compared to ED [16]. This difference was possibly due to iAUC considering fixed timings from 23:00 to 08:00 hours in their study, even though ED was eaten at 18:00 hours. We considered the iAUC from the pre-dinner value and for 10-hour post-dinner. Both 10H and 24H ABG values were reduced significantly by 12.39 mg/dl and 7.39 mg/dl, respectively. This is in agreement with previous studies [7,9,16,27] (Table 5). Night MAGE was reduced significantly in ED as compared to LD. Imai et al. showed a nonsignificant reduction in 24-hour MAGE; however, they did not calculate night MAGE separately. CV, or 24-hour MAGE, was not significantly different between LD and ED in our study [16].

**Table 5:** Studies assessing the change in ABG between LD and ED ABG, average blood glucose; ED, early dinner; LD, late dinner; OGTT, oral glucose tolerance tests; T2D, type 2 diabetes

Study	LD	ED	Study population	Duration of ABG (hours)	Reduction in ABG (mg/dl)	P-value
Sato et al. (2011) [9]	1.125	19:00	Healthy	23:00-06:00	4	<0.05
Imai et al. (2017) [16]	21:00	18:00	T2D	24 hours	10.98	<0.01
Gu et al. (2020) [7]	22:00	18:00	Healthy	17:00-12:00 the next day	6	<0.01
Garaulet et al. (2022) [27]	One hour before bedtime	Four hours before bedtime	Healthy	Two-hour OGTT	8.30%	0.022
This study	>22:00; average: 22:28	<20:00; average: 19:29	T2D	10-hour post-dinner	12.39	<0.002
				24 hours	7.39	0.036

hsCRP and IL-6 are well-known inflammatory markers known to influence cardiovascular outcomes, and meal-induced increases in these markers are attenuated by glycemic control [28]. We found nonsignificant reductions in these inflammatory markers between ED and LD. This contrasts with a previous study in T2D/prediabetes patients, which found significantly higher values of these markers among LD eaters than ED eaters [29]. However, the two groups were not matched for potential confounders in this cross-sectional study. In another study, five weeks of time-restricted feeding in prediabetics did not improve these inflammatory markers despite an 18-hour gap between dinner and breakfast the following day [23]. Thus, although, in our study, the markers started reducing after seven days of dinner time modification, these markers may take longer to show a more significant change.

Our study showed that despite the sudden change in dinner timings causing an initial increase in hunger at bedtime, as noted on the LMSS, it returned to baseline by Day 10. This may be attributed to the change in the leptin zenith during ED, as previously studied by Schoeller et al. [30].

One strength of our study is its practical, real-world nature. We assessed the effect of modifying only the dinner timings without modifying the patient’s other routine activities. We have also assessed the effect of crossover ED for a more extended period than numerous other studies, which have examined only the acute effects of a single early meal [9,16,17,27].

The practical nature of this study could also be a potential limitation. The patient was not given a standardized meal on the day of testing, as we wanted to assess the glycemic control of the patient’s usual dinner. Another limitation is the small sample size, which would require larger studies to validate the results. We also did not use actigraphy for sleep or physical activity timing. However, these limitations were minimized by keeping a record of a food diary, physical activity, sleep timings, and a blinded CGMS.

## Conclusions

A simple modification of preponing dinner in habitual late eaters with uncontrolled T2DM improves FPG, 10-hour post-dinner glucose excursions, and overall glycemic control in the short term. The likely mechanism is an improvement in IR, as evidenced by the reduction in HOMA-IR. Significant improvements in nighttime glycemic variability and lipid profile were also noted. However, inflammatory markers and markers of oxidative stress may take longer to show meaningful reductions.
